# Association between self-reported and objectively assessed physical functioning in the general population

**DOI:** 10.1038/s41598-024-64939-z

**Published:** 2024-07-14

**Authors:** Nicola Moser, Floran Sahiti, Götz Gelbrich, Vladimir Cejka, Fabian Kerwagen, Judith Albert, Stefan Frantz, Peter U. Heuschmann, Stefan Störk, Caroline Morbach

**Affiliations:** 1 Department Clinical Research and Epidemiology, Comprehensive Heart Failure Center, University Hospital Würzburg, Am Schwarzenberg 15, D–97078 Würzburg, Germany; 2 Department Medicine I, University Hospital Würzburg, Würzburg, Germany; 3 Institute of Clinical Epidemiology and Biometry, University of Würzburg, Würzburg, Germany; 4 Clinical Trial Center, University Hospital Würzburg, Würzburg, Germany

**Keywords:** Self-reported physical functioning, Self-reported health, Six-minute walk test, Reliable self-assessment, Physical fitness, Cardiology, Medical research, Epidemiology, Public health, Cardiovascular diseases, Health care

## Abstract

Knowledge about a patient’s physical fitness can aid in medical decision-making, but objective assessment can be challenging and time-consuming. We aimed to investigate the concordance of self-reported health status and physical functioning with the 6 minute walking distance (6MWD) as objective measure of physical performance. The prospective characteristics and course of heart failure stages A/B and determinants of progression (STAAB) cohort study iteratively follows a representative sample of residents of the city of Würzburg, Germany, aged 30–79 years, without a history of heart failure (HF). The 6MWD was measured in 2752 individuals (aged 58 ± 11 years, 51% women) from a population-based cohort under strictly standardized conditions. Self-reported health status and physical functioning were assessed from items of the short form 36 (SF-36). After the respective classification of self-reported health status and physical functioning into ‘good’, ‘moderate’, and ‘poor’, we determined the association of these categories with 6MWD by applying a generalized linear model adjusted for age and sex. Prevalence of self-reported good/moderate/poor general health and physical functioning was 41/52/7% and 45/48/7%, respectively. Mean 6MWD in the respective categories was 574 ± 70/534 ± 76/510 ± 87 m, and 574 ± 72/534 ± 73/490 ± 82 m, with significant sex-specific differences between all categories (all p < 0.001) as well as significant differences between the respective groups except for the categories ‘moderate’ and ‘poor’ health status in men. This cross-sectional analysis revealed a strong association between self-reported health status and physical functioning with the objective assessment of 6MWD, suggesting that physicians can rely on their patients’ respective answers. Nevertheless, sex-specific perception and attribution of general health and physical functioning deserve further in-depth investigation. Decision-making based on self-reported health requires prospective evaluation in population-based cohorts as well as adult inpatients.

## Introduction

“How are you today?” is a non-specific opening phrase frequently exchanged in a medical interview between physicians or health care providers and patients. The phrase addresses an individual’s current health perception and, if used as a general screening question, may reflect the self-reported health status^1–3^. Although the answer to this question might be regarded lacking in-depth information^2,4^, it showed prognostic utility^5^, even after adjusting for common risk factors, sociodemographic background^6^, and objective health measures such as biomarkers and comorbidities^4^. There are well-established tools to reliably assess self-reported health and physical functioning^7–11^, but the yield of self-reported responses has been a subject of debate, particularly regarding their ability to predict objective health outcomes^2,5^.

Further, evidence suggests that self-reported health carries significant prognostic information^12^. A meta-analysis showed that individuals with ‘poor’ self-reported health had a two-times higher risk of all-cause mortality than those with ‘excellent’ self-reported health^4^. Another large prospective cohort study reported a strong association between self-reported health and all-cause mortality risk over a 25 year follow-up period, with better self-reported health predicting better survival, irrespective of more objective measures of health^12^.

Objectively measured physical functioning provides specific and accurate information with reduced bias. However, it can be challenging, time-consuming, expensive, and not always feasible for all individuals. On the other hand, self-reported physical functioning emerges as a useful tool in clinical practice^13^. In contrast to objectively assessed physical fitness, self-reported physical functioning has been shown to be time-efficient and cost-effective^10^, also capturing patients’ perspectives. However, it may be prone to bias and might not always accurately reflect reality. Such regular assessment could help to detect a decline in the level of physical functioning and identify the need for further testing and potential interventions^14^.

Since most studies examining self-reported health and physical functioning focused on specific patient samples defined by selected categories of age or comorbidity^1,15,16^, studies among the general population, which would be of particular importance for general practitioners for example, are scarce. We, therefore, aimed to evaluate the association of self-reported health and self-reported physical functioning, respectively, with objective physical performance quantified by the 6 minute walking distance (6MWD) in a well-characterized population-based cohort with respect to potential sex-specific differences.

## Methods

### Study design, population, and recruitment

Details on the study design and primary results of the characteristics and course of heart failure stages A/B and determinants of progression (STAAB) cohort study have been described previously^17,18^. In brief, the prospective, population-based STAAB cohort study is a joint project of the department of clinical research and epidemiology of heart failure of the comprehensive heart failure center (CHFC) and the Institute of Clinical Epidemiology and Biometry (IKE-B) of the University Hospital and the University of Würzburg, Germany. The STAAB study aims to determine the prevalence of the early asymptomatic stages of heart failure A/B in a representative sample of 5000 residents of the City of Würzburg, Germany, and to prospectively investigate the determinants of progression into the symptomatic stages of heart failure. This study has been approved by the Ethics Committee of the Medical Faculty of the University of Würzburg (vote #98/13) and the data protection officer of the University and the University Hospital of Würzburg and complies with the Declaration of Helsinki. All study participants provided their written informed consent to participate in the study prior to any examination.

STAAB aimed to recruit a random sample of 5000 residents of the City of Würzburg (number of inhabitants; n = 124,297, as of 2011), stratified for sex (ratio women: men = 1:1) and the following age decades: 30–39/40–49/50–59/60–69/70–79 years (respective sampling ratio = 10:27:27:27:10). Individuals with pre-existing HF were excluded from the study. The baseline examination took place between December 2013 and October 2017^18^.

### Follow-up examination and selection criteria for current analyses

STAAB participants who had attended the baseline examination (n = 4965 participants) were invited by mail to a first follow-up examination. A total of 3901 individuals responded positively to the follow-up examination, which lasted from December 2017 to August 2021 (including a 15 month COVID-19 pandemic-induced break).

During the initial phase of the COVID-19 pandemic and during the periods when participation in the study was completely restricted, no follow-up visits were conducted. Although the vast majority of follow-up visits had already been performed, the pandemic affected the pace of the follow-up phase and consequently might have influenced the total number of participants attending the follow-up. Nonetheless, we identified no reason why the pandemic might have directly affected the present results. The follow-up visit took place at the joint survey unit of the CHFC and ICE-B and included anthropometric measures, electrocardiogram, echocardiography, physical examination, collection of blood for standard parameters and long-time storage, as well as questionnaires including the short form-36 interrogating self-reported generic quality of life^17,19^. All examinations were performed by trained staff according to standard operating procedures and documented in case report forms^17^.

Participants were included in the statistical analysis if they had completed the SF-36 questionnaire and had a valid 6MWD. The latter was assumed if the participant had no contraindications or logistic limitations to performing the 6 minute walking test (6MWT) and did not prematurely terminate the 6MWT. Absolute contraindications for the 6MWT were the presence of unstable angina pectoris or a previous myocardial infarction within the last 4 weeks. Relative contraindications were a resting heart rate above 120 beats per minute and/or a systolic blood pressure above 180 mmHg and/or a diastolic blood pressure above 100 mmHg. In addition, other reasons (e.g. orthopedic problems), according to the individual assessment of the study physician, could lead to the 6MWT not being performed. Furthermore, logistic factors (e.g. inadequate footwear or lack of staff) could result in the 6MWT not being performed.

### Six-minute walk test (6MWT)

The 6MWT was performed according to a standardized protocol using a 15 m test distance located in an undisturbed, straight, and flat indoor hallway. Each participant performed the 6MWT once and under the supervision of a trained staff member. Participants were instructed to cover as much ground as possible within six minutes, without running or jogging. They could slow down or stop if necessary but should resume walking as soon as possible. During the 6MWT, participants were encouraged verbally every 30 s to continue walking. Blood pressure and heart rate were assessed before and after the 6MWT. The 6MWD was documented in a case report form in meters^20^.

### Categorization of self-reported health status and self-reported physical functioning

Self-reported health status and self-reported physical functioning were assessed using selected questions from the short form 36 questionnaire (SF-36)^17^. The SF-36 is a widely used tool to measure health-related quality of life^19^. The SF-36 consists of eight separate domain scores, two of which describe ‘physical functioning’^19^ and ‘general health’^21^. For this analysis, four questions were selected; one question addressing the self-reported health status and three questions from the domain ‘physical functioning’.

**Figure 1:**
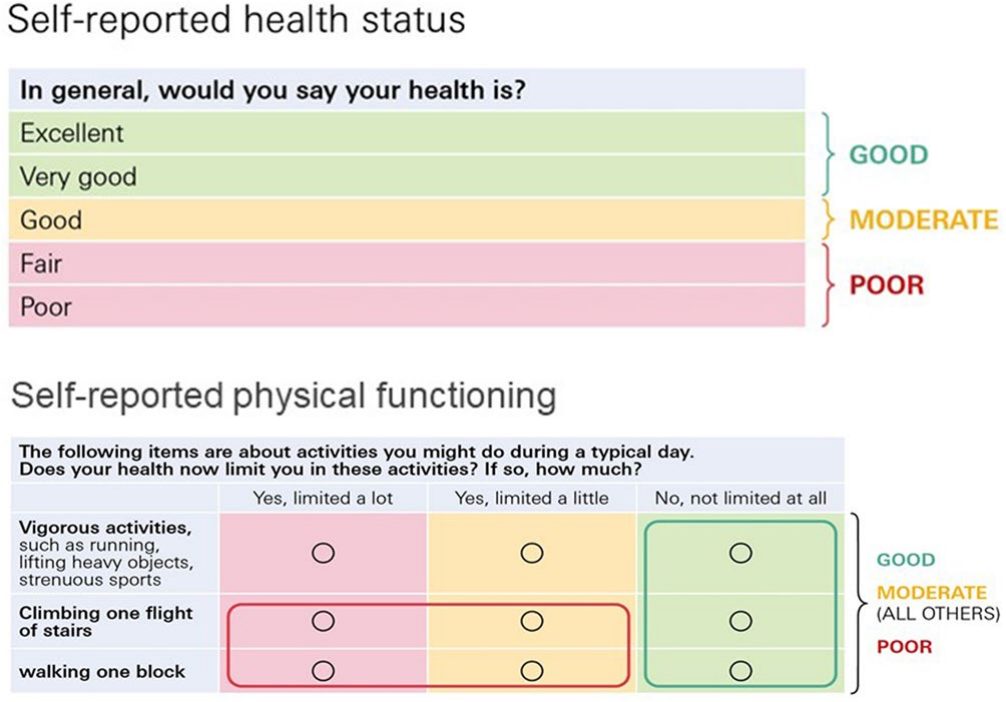
(**A**,**B**) Categorization of self-reported health status and self-reported physical functioning. Self-reported health status (short form-36, question #1) was re-categorized into ‘good’ (excellent and very good), ‘moderate’ (good), and ‘poor’ (fair and poor). Self-reported physical functioning (short form-36, questions #3, #7, and #11) was re-categorized into ‘poor’ (limited a lot or limited a little in climbing one flight of stairs and/or walking one street crossing; ≥ 1 ‘yes’ in the red box), ‘good’ (not limited at all; all ‘yes’ in the green box) and ‘moderate’ (all other combinations). The category ‘moderate’ served as the reference category both for self-reported health status and self-reported physical functioning.

The SF-36 question #1 on general health (“How would you describe your health status in general?”) has five answer options to choose from: ‘excellent’, ‘very good’, ‘good’, ‘fair’, and ‘poor’. To avoid very small group sizes in statistical analysis, three categories were formed from the five answer options: ‘good’ (excellent or very good) ‘moderate’ (good; in statistical analyses used as the reference category), and ‘poor’ (fair or poor). Similarly, self-reported physical functioning was categorized into three groups. Individuals who reported being slightly or severely impaired at ‘climbing one flight of stairs’ or ‘walking one block’ were assigned to the category ‘poor’. Individuals reporting no limitations in both of these as well as in vigorous physical activities were assigned to the category ‘good’. All other individuals were assigned to the category ‘moderate’, which served as the reference category in the statistical analyses. The re-categorization process is illustrated in Fig. 1A, B.

### Data analysis

All statistical analyses were performed using the SPSS Statistics (version 28.0). Categorical variables are presented as frequencies (percent), while continuous variables were described using mean (standard deviation) or median (quartiles). Comparisons of continuous variables between the three categories ‘good’, ‘moderate’, and ‘poor’ of self-reported health status and self-reported physical functioning, respectively, were performed using the *t*-test for independent samples and ANOVA. The homogeneity of variances was tested with the Levene’s test. Differences between categories were evaluated with the Scheffé test in case variances were equal, and with the Dunnett T3 test in case variances were unequal. Subject characteristics in each category are summarized in Tables 2 and 4.

The association of the three categories ‘good’, ‘moderate’, and ‘poor’ of self-reported health status and self-reported physical functioning, respectively, with the 6MWD was calculated using general linear models, adjusted for age and sex. A multiplicative interaction term was included to test for sex interactions, and in case of significance (p < 0.05), effects were reported separately for women and men (Table 3 and Supplemental Table 1). A p-value < 0.05 was considered significant.

## Results

Of the 4965 individuals (55 ± 12 years, 52% women) initially included in the STAAB cohort study^18^, 3901 individuals (58 ± 11 years, 52% women) attended the first follow-up examination. Of those, 2762 participants (58 ± 11 years, 51% women) had a valid 6MWD. Ten of them had incomplete SF-36 forms and were excluded from further analysis. Hence, 2752 participants (58 ± 11 years, 51% women; Table 1) were included in the statistical analyses (Supplemental Fig 1). The mean 6MWD for the total sample was 549 ± 77 m. Men walked a longer distance (558 ± 78 m) when compared to women (540 ± 76 m; p < 0.001).

**Table 1:** Characteristics of participants with a valid 6 minute walking distance and valid short form 36 questionnaire.

	Total N = 2752 (100%)	Men N = 1345 (48.9%)	Women N = 1407 (51.1%)
Age [years]	58 (11)	58 (11)	57 (11)
Body height [cm]	171 (9)	178 (7)	165 (6)
Body mass index [kg/m^2^]	26.2 (4.5)	27.0 (4.3)	25.4 (4.7)
Systolic blood pressure [mmHg]	130 (17)	134 (16)	126 (17)
Diastolic blood pressure [mmHg]	77 (10)	79 (10)	75 (9)
Resting heart rate [min^−1^]	67 (10)	66 (10)	68 (9)
Comorbidities			
Hypertension^**÷**^	1295 (47.0)	737 (54.8)	558 (39.7)
Dyslipidaemia^**§**^	398 (14.5)	226 (16.8)	172 (12.2)
Cardiovascular disease^+^	153 (5.5)	111 (8.3)	42 (2.9)
Diabetes mellitus^**¥**^	165 (5.9)	108 (8.0)	57 (4.1)
Obesity^#^	463 (16.8)	249 (18.5)	214 (15.2)
Smoker^	1416 (51.4)	768 (57.1)	648 (46.1)
Laboratory analysis			
eGFR [mL/min/1.73 m^2^]	82 (14)	82 (14)	81 (14)
Haemoglobin [g/dL]	14.1 (1.2)	14.8 (1.0)	13.4 (0.9)
LDL cholesterol [mg/dL]	117 (34)	116 (33)	117 (34)
HDL cholesterol [mg/dL]	60 (50, 73)	53 (44, 62)	68 (59, 81)
Triglycerides [md/dL]	95 (70, 137)	106 (78, 154)	86 (66, 122)
HbA1c [%]	5.5 (0.5)	5.6 (0.6)	5.5 (0.5)
Fasting glucose [mmol/L]	5.2 (1.0)	5.3 (1.1)	5.1 (1.0)

Data are presented as mean (standard deviation), median (quartiles), or n (%), as appropriate.

*eGFR* estimated glomerular filtration rate, *LDL* low-density lipoprotein, *HDL* high-density lipoprotein, *HbA1c* glycosylated haemoglobin A1c.

÷ Hypertension: blood pressure ≥ 140/90 mmHg or taking antihypertensive pharmacotherapy.

^§^Dyslipidaemia: LDL cholesterol ≥ 190 mg/dl or taking a lipid-modifying drug.

+ Cardiovascular disease was self-reported and inquired the terms or respective descriptors of: “cardiovascular disease”, “myocardial infarction”, “percutaneous transluminal coronary angioplasty or stent”, “peripheral arterial disease”, and “stroke”.

¥Diabetes mellitus: HbA1c > 6.5% or taking a blood glucose lowering drug.

^#^Obesity: body mass index > 30 kg/m^2^.

^Smoker: active smoker or former smoker.

### Self-reported health status

Table 2 summarizes the characteristics of the participants per category of self-reported health status: 52% of the participants were assigned to the category ‘moderate’, 41% to the category ‘good’, and 7% to the category ‘poor’. The percentage of women and the mean age were highest in the category ‘poor’. The mean 6MWD was highest in the category ‘good’ and lowest in the category ‘poor’. 6MWD differed significantly between all categories (all p < 0.001), except for categories ‘moderate’ and ‘poor’ amongst men (p = 0.837).

**Table 2:** Subject characteristics according to the three categories of self-reported health status.

	Self-reported health status		
	Good	Moderate	Poor
Number (% of total sample)	1140 (41.4)	1425 (51.8)	187 (6.8)
Women; n (% of category)	530 (46.5)	769 (54.0)	108 (57.8)
Men; n (% of category)	610 (53.5)	656 (46.0)	79 (42.2)
Age [years]	54 (11)	60 (11)	61 (10)
Women	54 (11)	59 (11)	61 (11)
Men	55 (11)	60 (11)	61 (9)
6MWD [m]	574 (70)	534 (76)	510 (87)
Women	565 (67)	529 (74)	495 (88)
Men	582 (71)	539 (78)	532 (82)

Data are n (%) or mean (standard deviation).

*6MWD* 6 minute walking distance.

**Figure 2:**
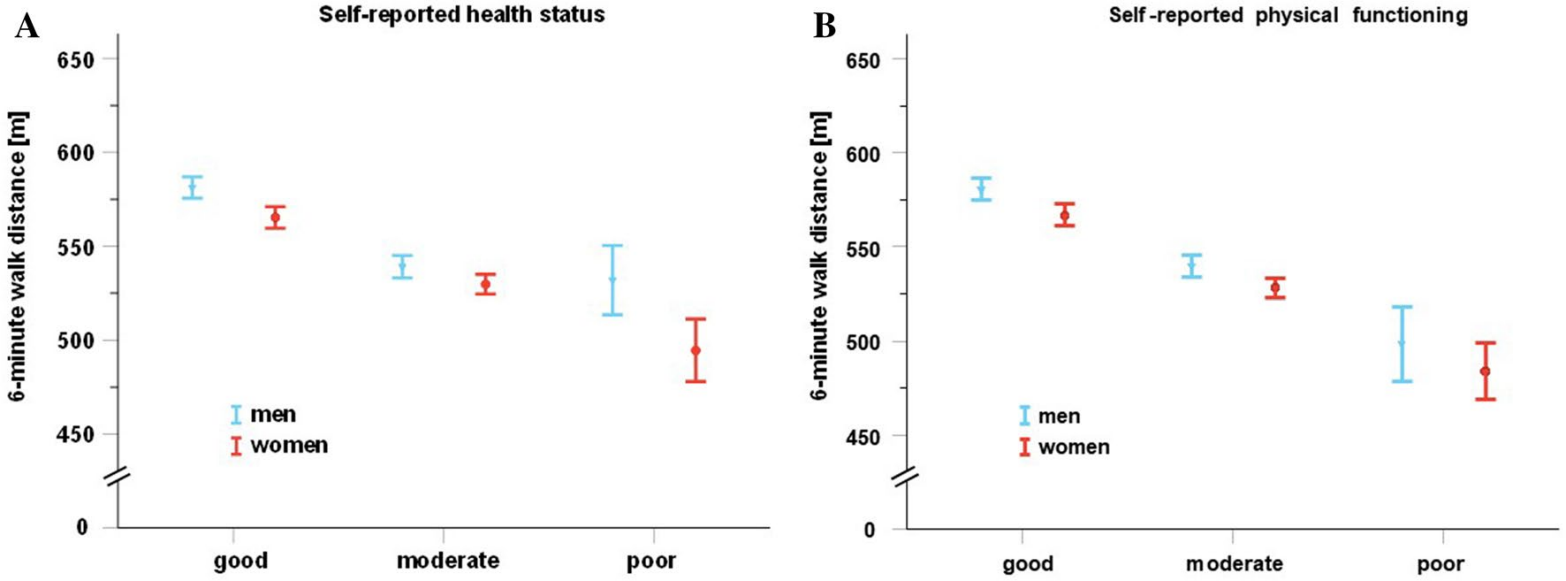
(**A**) Sex-specific 6 min walk distance (95% confidence interval) according to the three categories of self-reported health status. (**B**) Sex-specific 6 minute walking distance (95% confidence interval) according to the three categories of self-reported physical functioning.

The results of the general linear model (Table 3) showed that women in the category ‘good’ walked about 11 m further than those in the reference category ‘moderate’. The distance walked by women in the category ‘poor’ was about 20 m shorter than by women in the reference category ‘moderate’. Men in the category ‘good’ walked about 20 m further than men in the reference category ‘moderate’. Of note, amongst men, there was no significant difference in walking distance between the categories ‘moderate’ and ‘poor’. Figure 2A illustrates these results.

**Table 3:** Difference in 6 minute walking distance (= effect estimator) of subjects in the categories “good” and “poor” of self-reported health status with respect to the reference category “moderate”.

	Effect estimate [m] (95% confidence interval)*	P-value for estimate	P-value for interaction with sex
Good			0.018
Women	+ 11.4 (+ 3.8, + 19.1)	0.003	
Men	+ 19.7 (+ 12.1, + 27.3)	< 0.001	
Poor			
Women	− 20.1 (− 33, − 6.6)	0.004	
Men	+ 7.1 (− 8.4, + 22.7)	0.369	

*with respect to the reference category ‘moderate’.

### Self-reported physical functioning

Table 4 summarizes the characteristics of the individuals per category of self-reported physical functioning: 48% of individuals were assigned to the category ‘moderate’, 45% to the category ‘good’, and 7% to the category ‘poor’. The percentage of women and the mean age were again highest in the category ‘poor’. The mean 6MWD was highest in the category ‘good’ and lowest in the category ‘poor’. The mean 6MWD differed significantly (p < 0.001) between all categories, both in women and men.

**Table 4:** Subject characteristics according to the three categories of self-reported physical functioning.

	Self-reported physical functioning		
	Good	Moderate	Poor
Number (% of total sample)	1237 (45.0)	1327 (48.2)	188 (6.8)
Women; n (% of category)	574 (46.4)	718 (54.1)	115 (61.2)
Men; n (% of category)	663 (53.6)	609 (45.9)	73 (38.8)
Age [years]	54 (11)	60 (10)	64 (10)
Women	53 (11)	59 (10)	64 (10)
Men	54 (11)	61 (10)	63 (10)
6MWD [m]	574 (72)	534 (73)	490 (82)
Women	567 (70)	528 (71)	484 (81)
Men	581 (72)	540 (75)	498 (84)

Data are n (%) or mean (standard deviation).

*6MWD* 6 minute walking distance.

The results of the general linear model (Supplemental Table 1) indicated consistent effects in both sexes. Subjects in the category ‘good’ walked 15 m further than those in the reference category ‘moderate’. Those in the category ‘poor’ walked a 24 m shorter distance than those in the reference category ‘moderate’. Figure 2B illustrates these findings.

## Discussion

The important question, of whether the statements of persons regarding their general health status and physical functioning are reliable, has been addressed in selected groups of patients, but valid data from the general population are lacking so far. The current data aimed to narrow this knowledge gap by investigating a well-characterized population-based sample. Our results, based on cross-sectional data, reveal a strong relationship between self-reported measures of health status and physical functioning with objective physical performance as assessed by the 6MWD. While women showed a consistently shorter 6MWD with lower self-reported health status, the 6MWD of men reporting ‘moderate’ and ‘poor’ health status was similar, suggesting that men might consider other factors beyond physical performance when reporting a ‘poor’ health status. Better self-reported physical functioning was consistently associated with longer 6MWD both in women and men.

Objectively measured physical functioning provides specific and accurate information with reduced bias. On the other hand, self-reported physical functioning might cover a longer time span and a larger variety of physical activities and therefore be more representative than a singular assessment. With respect to self-reported health status, previous studies in diseased populations demonstrated a moderate correlation with the 6MWD, e.g. in patients investigated after an elective colon resection surgery (r = 0.43)^22^, in patients with heart failure (r = 0.34)^23^, and weak correlation in patients studied during cardiac rehabilitation (r = 0.21)^24^. In our sample from the general population, we observed significant differences in the 6MWD between all three categories of self-reported health status in women. Yet, in men, there was no difference between the categories ‘moderate’ and ‘poor’. This is consistent with a study in older people without chronic illnesses, where self-reported health status showed a significant moderate correlation with the 6MWD only in women (r = 0.38)^25^. It is conceivable that the very question addressing health status is differently perceived and interpreted by women versus men^25^. For instance, earlier research indicated that men in mid-life and older age tended to assign greater importance to physical impairments and adverse health behaviors when assessing their health when compared to women^26^. Previous studies have shown a relationship between low self-reported health status and diabetes mellitus, increased BMI, smoking, limited social support, unemployment, being unmarried, low social support, and little physical exercise^27^. This suggests that individuals rely on these factors when assessing their health. Differential weights applied to these factors might be the reason why the question about the general health status is interpreted and thus answered differently by men and women, and thus does not correlate equally in both sexes with results from the 6MWD.

Our results further show significant differences in the 6MWD between all three categories of self-reported physical functioning in both women and men. This is in agreement with prior studies on patient populations that found a moderate to strong association between physical functioning and the 6MWD^20,23–25,28^. In the present work, a mere 7% of participants fell into the ‘poor’ category concerning self-reported health and physical functioning. This distribution most likely might be attributed to the study’s design, including a population-based sample of individuals without heart failure. Further, the percentage of women in the category ‘poor’ of both self-reported health status and self-reported physical functioning was higher than the percentage of men. This finding is consistent with previous studies in which women also rated their health status and physical functioning worse than men^29^. Women’s generally longer lifespan does not automatically translate into healthier years of life, as women are more likely than men to suffer from chronic, non-lethal diseases (especially arthritis and depression) at any age^30,31^. In addition, the decline of physical functioning with aging tends to occur faster in women compared to men^13^. Furthermore, cardiovascular disease in women, despite being the leading cause of death in both sexes, continues to receive inadequate attention in terms of research, awareness, diagnosis, and treatment^32^ and therefore likely remains underdiagnosed and undertreated in women. All these factors might contribute to our finding of a higher prevalence of women in the category ‘poor’ of both self-reported health status and self-reported physical functioning and remain subject to further investigation.

The mean difference in 6MWD observed between the respective categories of general health and physical functioning might seem modest at face value. In patient cohorts, a change of about 30 m has been generally accepted as clinically relevant with respect to patient-related outcome measures^33–35^. However, evidence referring to the association of the minimal clinically meaningful difference in 6MWD with quality of life, i.e. focusing on what is clinically meaningful from the patient’s perspective, emerged only recently. A systematic review including six studies of patients with different pathologies revealed a change of 14–35 m as clinically meaningful^36^. Relating 6MWD to quality of life, Henricson et al.^37^ found a differential result in patients with muscular dystrophy: at lower levels of function, smaller increases in 6MWD resulted in a meaningful change in the quality of life, while at higher levels of function, larger increases were necessary to achieve such change. In patients with heart failure, Kahn et al.^38^ reported that an increase of 14 m in 6MWD over a period of 12 weeks corresponded to a meaningful improvement in exercise capacity, while a meaningful worsening was associated with a decrease of 31 m in 6MWD or more. Of note, respective data on a population level are lacking so far. Comparing groups of substantial size from the general population, the current investigation adds that differences in 6MWD of 11–24 m identified three categories of self-reported general health and physical functioning, respectively, suggesting that differences of this magnitude might be of clinical relevance. To corroborate this hypothesis, the determination of a minimal clinically relevant change over time in the general population remains the subject of future research.

### Strengths and limitations

Self-reported clinical information has various recognized constraints, particularly in relation to cultural variances^39^, the presence of bias such as social desirability^1^, and the potential for misinterpreting questions^39^. Nevertheless, we used a validated tool in a standardized way to assess self-reported health status and physical functioning, respectively, to achieve the highest reliability of study results. Further, we performed the 6MWT under strictly standardized conditions to minimize measurement variability and bias. A further strength of this study is that it was performed in a well-characterized representative sample of the general population. We utilized the 6MWD as the primary and most reliable outcome measure of the 6MWT. We acknowledge that other estimates, e.g. peakVO2, might also be derived from the 6MWT, although their utility is less firmly established. Further, future research and long-term observation will inform on measures like sensitivity to change and construct validity in the general population as well as on a potential prognostic yield.

### Implications for practice and research

Healthcare providers often ask patients to rate their health status as part of the initial assessment. However, when it comes to making clinical decisions, physicians and health professionals exhibit discrepancies when it comes to subjective and objective health measures, often presuming that subjective measures are less reliable and more susceptible to contextual influences than objective ones^40^. Nonetheless, the strong association found in the present study between objective physical performance and self-reported health status or physical functioning implies that self-reported statements are indeed reliable. This knowledge can however be used to integrate self-reported information on general health status and physical functioning into clinical practice. Future controlled studies are needed to investigate, whether questions on self-reported health status and self-reported physical functioning can serve as valuable screening tools helping to identify individuals at risk of developing health issues and to target interventions accordingly.

## Conclusion

This current study was conducted in a well-characterized population-based sample and demonstrated an association between SF-36-derived self-reported health status and physical functioning, respectively, with objective physical performance measured by the 6MWD. Based on these results, it can be inferred that self-reported health status and self-reported physical functioning are reliable indicators of objective health status and may function as a global assessment tool of health status in the general population. Self-reported health status and self-reported physical functioning might serve as a helpful alternative when objective data are not accessible and could be suitable screening tools to identify patients at risk.

### Supplementary Information

Supplementary Information.

## Data Availability

The data that support the findings of this study are not openly available due to reasons of sensitivity and are available from the corresponding author upon reasonable request.

## Abbreviations

6MWD: Six-minute walking distance
6MWT: Six-minute walking test
STAAB: Characteristics and course of heart failure stages A/B and determinants of progression
