# Supplementary material for: Association between self-reported and objectively assessed physical functioning in the general population
Source: Sci Rep. 2024 Jul 14;14:16236. doi: 10.1038/s41598-024-64939-z (PMC11247090; doi:10.1038/s41598-024-64939-z)
Supplement: Supplementary file 1 — Supplementary Information. [file 41598_2024_64939_MOESM1_ESM.pdf]

Supplemental Figure 1. Study consort

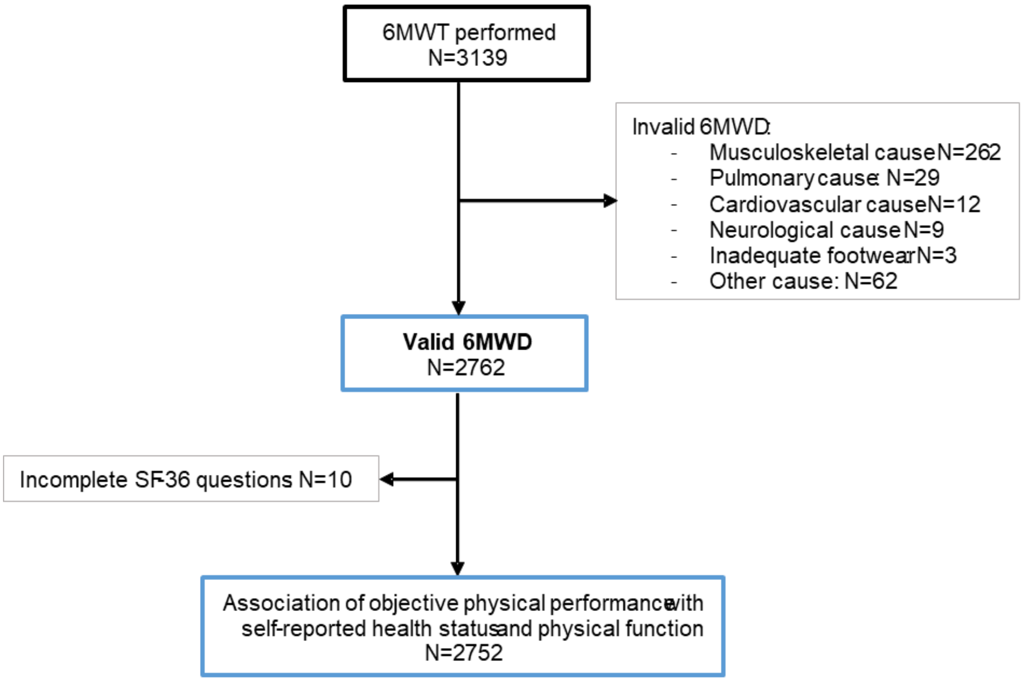

STAAB = Population-based *Characteristics and Course of Heart Failure Stages A-B and Determinants of Progression* Cohort Study; 6MWT = 6-minute walk test, 6MWD = 6-minute walk distance; SF-36 = Short Form 36.

Supplemental Table 1: Difference in six-minute walk distance (= effect estimator) of subjects in the categories "good" and "poor" of self-reported physical fitness, with respect to the reference category "moderate".

	**Effect estimate [m] (95% confidence interval)**	**P-value for estimate**	**P-value for interaction with sex**
**Good**	+14.5 (+6.9, +22.1)	<0.001	0.896
**Poor**	-24.3 (-37.6, -11.1)	<0.001	

*with respect to the reference category 'moderate'
